# iHIVARNA phase IIa, a randomized, placebo-controlled, double-blinded trial to evaluate the safety and immunogenicity of iHIVARNA-01 in chronically HIV-infected patients under stable combined antiretroviral therapy

**DOI:** 10.1186/s13063-019-3409-1

**Published:** 2019-06-17

**Authors:** Wesley de Jong, Joeri Aerts, Sabine Allard, Christian Brander, Jozefien Buyze, Eric Florence, Eric van Gorp, Guido Vanham, Lorna Leal, Beatriz Mothe, Kris Thielemans, Montse Plana, Félipe Garcia, Rob Gruters, on behalf of the iHIVARNA consortium, F. G., José M. Gatell, Joan-Albert Arnaiz, M. P., L. L., Albert Guardo, Maria José Maleno, G. V., E. F., Pieter Pannus, J. B., Leo Heyndrickx, K. T., Joeri Aerts, S. A., Patrick Tjok, C. B., B. M., J. Martinez-Picado, Alex Olvera, Miriam Rosas, Maria Salgado, Sara Moron, Jose Moltó, Miriam López, R. G., Marion Koopmans, W. J., Patrick Boers, Rachel Scheuer, Cynthia Lungu, Carlo Heirman, Sonja Van Meirvenne, Anna Graupera, Ángel Honrado

**Affiliations:** 1 Department of Viroscience, Erasmus MC, Room Ee-1726, P.O. Box 2040, 3000 CA Rotterdam, The Netherlands; 2 Laboratory of Molecular and Cellular Therapy, Vrije Universiteit Brussel, Brussels, Belgium; 3 Department of Internal Medicine and Infectious Diseases, Universitair Ziekenhuis Brussel, Brussels, Belgium; 4 Infectious Diseases Unit, IrsiCaixa AIDS Research Institute, Hospital Germans Trias i Pujol, Badalona, Spain; 5 Institució Catalana de Recerca i Estudis Avançats (ICREA), Barcelona, Spain; 6 AELIX Therapeutics, Parc Científic de Barcelona, Barcelona, Spain; 7 University of Vic – Central University of Catalonia (UVic-UCC), Vic, Spain; 8 Virology Unit, Department of Biomedical Sciences, Institute of Tropical Medicine and, Antwerp, Belgium; 9 Department of Biomedical Sciences, University of Antwerp, Antwerp, Belgium; 10 Institut d’Investigacions Biomèdiques August Pi i Sunyer (IDIBAPS), Villarroel, 170, 08036 Barcelona, Spain; 11 Infectious Diseases Unit, Hospital Clínic, Villarroel, 170, 08036 Barcelona, Spain

**Keywords:** HIV-1, Therapeutic vaccine, Functional cure, Immunotherapy, Reservoir, Lymph node, TriMix, Antigen-presenting cell

## Abstract

**Background:**

HIV therapeutic vaccination aims to improve the immune responses against HIV in order to control viral replication without the need for combined antiretroviral therapy (cART). iHIVARNA-01 is a novel vaccine combining mRNA delivery and T-cell immunogen (HTI) based on conserved targets of effective antiviral T-cell responses. In addition, it holds adequate stimuli required for activating antigen presenting cells (APC)s and co-activating specific T-cells (TriMix), including human CD40L, constitutively active TLR4 (caTLR4) and CD70. We propose that in-vivo targeting of dendritic cells (DCs) by direct administration of a HIV mRNA encoding these immune modulating proteins might be an attractive alternative to target DCs in vitro.

**Methods/design:**

This is a phase-IIa, randomized, double-blinded, placebo-controlled, multicenter study in chronically HIV-1 infected patients under stable cART. One of the three study arms is randomly allocated to subjects. Three vaccinations with either HIVACAT T-cell immunogen (HTI)-TriMix (iHIVARNA-01), TriMix or water for injection (WFI) (weeks 0, 2 and 4) are administered by intranodal injection in the inguinal region. Two weeks after the last immunization (week 6) cART is stopped for 12 weeks. The two primary endpoints are: (1) safety and tolerability of intranodal iHIVARNA-01 vaccination compared with TriMix or WFI and (2) induced immunogenicity, i.e., increase in the frequency of HIV-specific T-cell responses between baseline, week 6 and 12 weeks after treatment interruption in iHIVARNA-01-treated patients as compared to the control groups, immunized with TriMix-mRNA or WFI measured by an IFNγ ELISPOT assay. Secondary endpoints include the evaluation of time to viral rebound, plasma viral load (pVL) at w18, the proportion of patients with control of viral load, induction of T-cell responses to new HIV epitopes, polyfunctionality of HIV-specific T-cells, CD8+ T-cell in-vitro HIV suppressive capacity, the effect on viral reservoir (measured by proviral DNA and cell-associated RNA), assessment of viral immune escape by mutation and mRNA expression profiles of host immune genes.

**Discussion:**

This trial aims to direct target DC in situ with mRNA encoding HTI and TriMix for co-stimulation. Intranodal injection circumvents laborious DC isolation and handling in the laboratory. The trial extends on the safety results of a phase-I dose-escalating trial. This candidate vaccine could complement or even replace cART for chronic HIV infection and could be applicable to improve the care and cost of HIV infection.

**Trial registration:**

EudraCT 2016-002724-83 (22 September 2016); ClinicalTrials.gov, ID: NCT02888756. Registered on 23 August 2016.

**Electronic supplementary material:**

The online version of this article (10.1186/s13063-019-3409-1) contains supplementary material, which is available to authorized users.

## Background

Worldwide, an estimated 37 million people are infected with the human immunodeficiency virus (HIV) [1]. HIV is a single-stranded, positive-sense ribonucleic acid (RNA) virus of the genus *Lentivirus*. The virus infects human CD4+ T-cells but also various mononuclear myeloid cells, including dendritic cells (DCs), monocytes/macrophages. It integrates into the host genome after reverse transcription to double-stranded deoxyribonucleic acid (DNA). In untreated patients, the infection progresses to acquired immunodeficiency syndrome (AIDS) – as characterized by a decline in CD4+ T-cells [2]. In 2015, an estimated 17.5 million people worldwide received combined antiretroviral therapy (cART) [1]. Despite being very effective at stabilizing the HIV infection and preventing virus transmission, cART has several disadvantages, including short- and long-term side effects. The therapy cannot eradicate HIV and, therefore, has to be maintained for life [3]. As a consequence, HIV therapy is costly to maintain. Furthermore, both therapy distribution and adherence are a challenge for patients and physicians – in both developing as well as developed countries [4]. Therefore, there is a clear unmet need for cost-effective and viable non-drug-based treatment in HIV-infected individuals.

Therapeutic vaccination has emerged as a strategy which aims to improve the immune responses against HIV in order to control viral replication without the need for cART [5]. To date, numerous immunogens, administration routes and vaccination strategies have been employed, so far with only limited success [5–8]. Garcia and colleagues obtained promising results in a strategy known as “ex-vivo modification of DCs” [8]. HIV-infected patients were administered autologous DCs pulsed with whole, inactivated HIV particles. This DC-based vaccine offers a promising strategy by using the ability of DCs to take up and present antigens to CD4+ and CD8+ T-cells. The immune responses act against viral replication by lysing HIV-infected cells. However, a functional cure (i.e., undetectable HIV plasma viral load, i.e., < 50 copies/mL) was not achieved. Furthermore, ex-vivo modification of DCs is a technical and labor-intense strategy.

The iHIVARNA-01 is intended for therapeutic vaccination of HIV-1-infected patients. It is a combination of mRNA sequences that aim to provide (1) an effective HIV immunogen to induce T-cell responses against relatively conserved, vulnerable portions of the virus, referred to as HIVACAT T-cell immunogen (HTI) as well as (2) adequate stimuli required for activating APCs and co-activating specific T-cells (TriMix). The former is designed to induce T-cell responses with specificities associated with superior HIV control in vivo and covering a wide range of HLA class I- and class II-restricted, HIV-derived epitopes [9]. The TriMix portion provides activation molecules to enhance T-cell activation and antigen presentation by DCs. The mRNA sequences correspond to human CD40L, constitutively active TLR4 (caTLR4) and CD70 [10]. iHIVARNA-01 investigational product (refer to section “Methods/design”) is the result of the iHIVARNA phase-1 clinical trial. In 2016, 21 HIV-infected volunteers were recruited in Hospital Clínic in Barcelona. Subjects received mixed ratios of TriMix:HTI and TriMix solutions, up to 1200 μg (900 μg HTI + 300 μg TriMix).^1^

## Methods/design

### Primary and secondary objectives

This study will evaluate the safety, tolerability and immunogenicity of iHIVARNA-01. The primary objectives are to evaluate:

- the safety and tolerability of intranodal iHIVARNA-01 vaccination compared with TriMix-mRNA or placebo, focusing on the nature, frequency and severity of local adverse events (pain, cutaneous reactions including induration) and systemic adverse events (temperature, chills, headache, nausea, vomiting, malaise and myalgia)
- the immunogenicity of an immunization schedule with HTI-TriMix (iHIVARNA-01) to increase the frequency of HTI-specific T-cell responses between baseline and 2 and 14 weeks after the last injection (i.e., weeks 6 and 18 of the study) as compared to the control groups, immunized with TriMix-mRNA only or placebo

The secondary objectives are to evaluate:

- the magnitude and the kinetics of the HIV-specific CD4+ and CD8+ T-cell responses generated by the immunization schedule in the three groups by two methods (ELISPOT, intra-cellular cytokine staining – ICS) at baseline and at weeks 6 and 30
- the ability of the immunization schedules to prolong time until viral rebound after discontinuation at week 6 as compared to control groups TriMix or placebo
- the suppressive effect on plasma viral load in vivo after analytical treatment interruption (ATI) from week 6 to week 18 compared to two control groups, receiving TriMix only or placebo
- the proportion of patients with control of viral load below detectable level 12 and 24 weeks after start of ATI (functional cure)
- the percentage of patients who generate a vaccine-induced immune response to new HIV epitopes present in the HTI sequence
- induction or enhancement of the CD8+ T-cell in-vitro HIV-suppressive capacity
- the effect on reservoir as measured by proviral DNA and the cell-associated viral RNA (unspliced and multiple spliced viral RNA)
- viral immune escape by sequencing of HIV and to conduct sieve-effect analyses in rebounding virus after cART interruption
- host protein mRNA expression profiles in whole blood at baseline and week 6 and week 18
- storage of fecal samples for future exploratory analysis of gut microbiota composition and diversity

### Study design, treatment arms and stages

This is a phase-IIa, randomized, double-blinded, placebo-controlled outpatient-clinic-based multicenter study in chronically HIV-1 infected patients under stable cART. Enrolled and randomized patients will be treated in one of the three study arms, comprising two stages.

Stage I consists of an immunization period from week 0 to week 6, while on cART. The immunization is done by an intranodal injection in a prominent lymph node in the inguinal region. The study arm (verum – HTI-TriMix (iHIVARNA-01) *n* = 40, control – TriMix *n* = 15 and placebo – water for injection (WFI) *n* = 15) is randomly allocated and stratified per center. An injection is given at weeks 0, 2 and 4. Stage II consists of the post-immunization and analytical treatment interruption (ATI) period (cART is stopped) and lasts from week 6 to week 18. Patients with an undetectable viral load at week 18 will remain off cART up to week 30. Study end is at week 30. In case a patient still has an undetectable (< 50 copies/mL) viral load, they may continue cART interruption in consultation with the treating physician. cART can be re-initiated for medical reasons at any time and/or if the CD4+ T-cell counts drop below 50% of baseline or below 350 cells/mL.

**Fig. 1:**
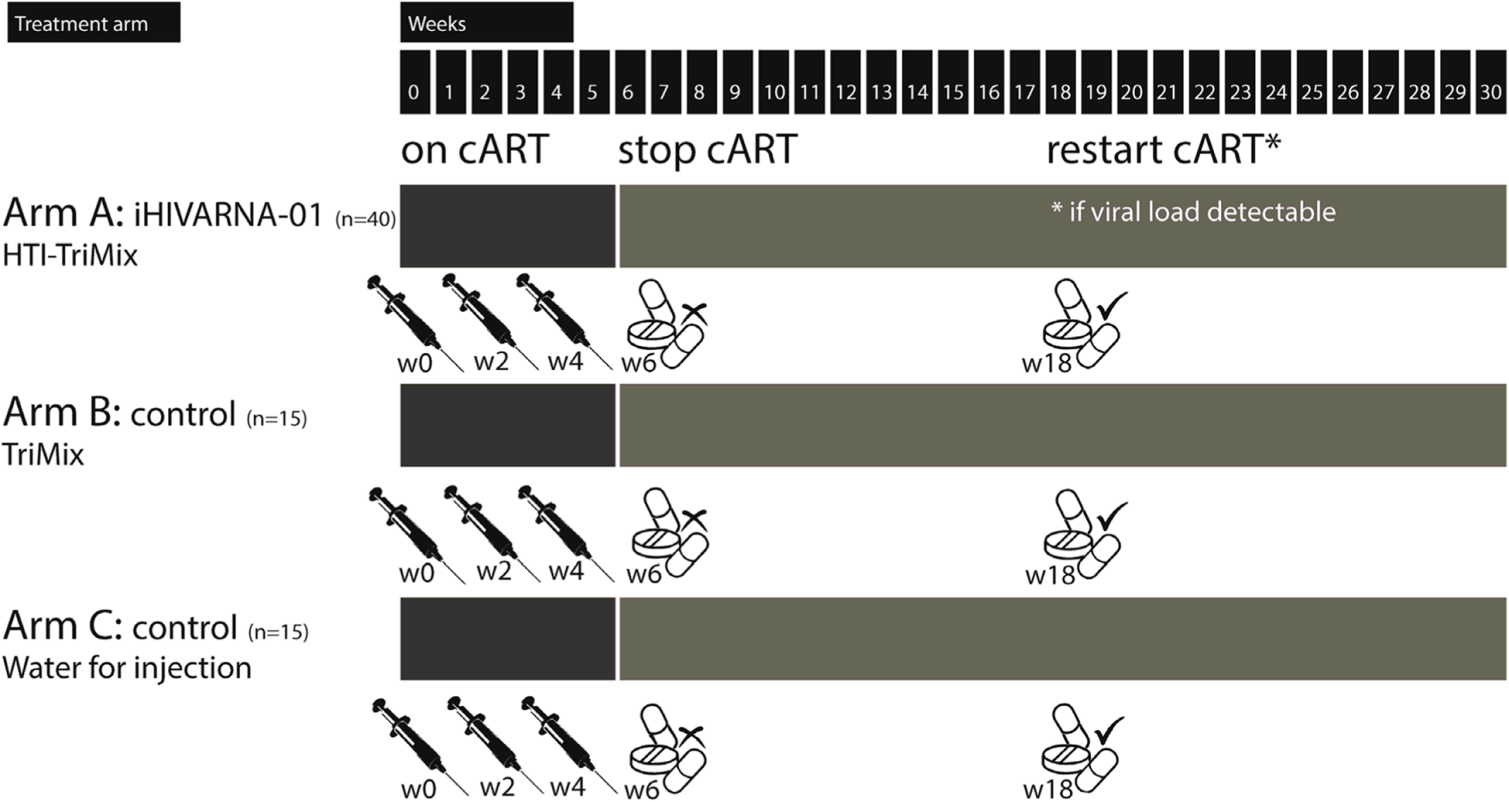
Overview of the study period, interventions and assessments

Refer to Fig. 1 for a visual overview of the stages and treatment arms.

### Inclusion and exclusion criteria

To be eligible to participate, a patient must meet all of the following criteria (inclusion criteria):

1. Age ≥ 18 years of age
2. Voluntarily signed informed consent
3. Proven HIV-1 infection (with documented antibodies against HIV-1 and a detectable plasma HIV-1 RNA before initiation of therapy)
4. On stable treatment with cART regimen (antiretroviral therapy consisting of at least three registered antiretroviral agents) for at least 3 years
5. Nadir CD4+ ≥ 350 cells/μL (up to two occasional determinations ≤ 350 cells/μL are allowed)
6. Current CD4+ cell count ≥ 450 cells/μL
7. HIV-RNA below 50 copies/mL in the last 6 months prior to randomization, during at least two measurements (occasional so called “blips” ≤ 500 copies/mL are permitted)
8. If sexually active, willing to use a reliable method of reducing the risk of transmission to their sexual partners during treatment interruption (including PrEP)
  - For heterosexually active women, using an effective method of contraception with partner (combined oral contraceptive pill; injectable or implanted contraceptive; IUD/IUS; consistent record with condoms; physiological or anatomical sterility (in self or partner) from 14 days prior to the first vaccination until 4 months after the last vaccination
  - For heterosexually active men, using an effective method of contraception with their partner from the first day of vaccination until 4 months after the last vaccination

Patients who meet any of the following criteria will be excluded from participating in the study (exclusion criteria):

1. Treatment with a non-cART regimen prior to the cART regimen
2. Previous failure to antiretroviral and/or mutations conferring genotypic resistance to antiretroviral therapy
3. Non-subtype B HIV infection
4. Active hepatitis B virus and/or hepatitis C virus co-infection
5. History of a CDC class C event [11];
6. Pregnant woman (screened with a positive pregnancy test), lactating or intending to become pregnant during the study
7. History of malignancy ≤ 30 days (extended period on the clinical assessment of the investigator) prior to screening
8. Active infection with fever (38 °C or above) ≤ 10 days of screening and/or first vaccination
9. Therapy with immunomodulatory agents (e.g., systemic corticosteroids), including cytokines (e.g., IL-2), immunoglobulins and/or cytostatic chemotherapy ≤ 90 days prior to screening. This does not include seasonal influenza, hepatitis B and/or other travel-related vaccines
10. Congenital, acquired or induced coagulation disorders, such as thrombocytopenia (thrombocytes < 150 × 10^9^/L) and/or current use of anti-coagulant medication (e.g., coumarins, inhibitors of Xa); usage of nonsteroidal anti-inflammatory drugs (NSAIDs) (including acetylsalicylic acid) is allowed; however, it is advised to interrupt therapy 10 days ahead of vaccination
11. Usage of any investigational drug ≤ 90 days prior to study entry
12. An employee of the investigator or study site, with direct involvement in the proposed study or other studies under the direction of that investigator or study site, or is a family member of an employee or the investigator
13. Any other condition, which, in the opinion of the investigator, may interfere with the evaluation of the study objectives

### Study procedures: informed consent and screening

**Fig. 2:**
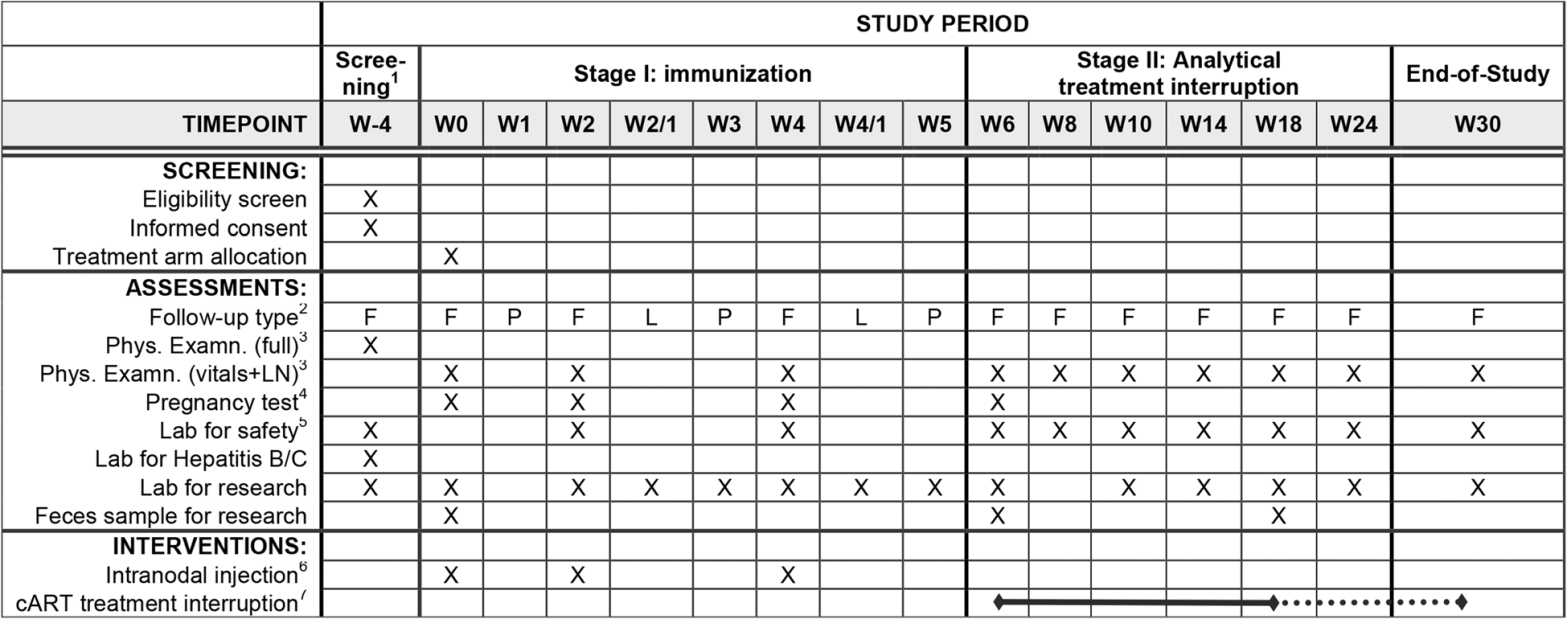
Screening, assessments and interventions schedule for iHIVARNA phase IIa. ^1^ screening at most 8 weeks prior to enrollment at W0. Interval of 4 to 2 weeks is advised and optional consideration period of up to 2 weeks is offered. ^2^ *F* full visit, *L* laboratory alone, *P* phone call. ^3^ full physical examination vs. vitals/lymph nodes alone. A full physical examination must be performed during follow-up when clinically indicated. ^4^ applicable to female subjects (reproductive age and not anatomically sterilized). Intranodal injection and treatment interruption is only allowed after a negative pregnancy test. ^5^ laboratory tests for safety includes hemoglobin, hematocrit, leucocytes, antibodies. Neutrophil count, thrombocytes, creatinine, aspartate aminotransferase (ASAT), alanine transaminase (ALAT), gamma-glutamyl transpeptidase (GGT), alkaline phosphatase (ALP), total bilirubin, amylase (or lipase), creatine kinase (CK, known as creatine phosphokinase), glucose, CD4+ and CD8+ cells and HIV viral load. ^6^ follow-up of vital signs (RR/pulse) at 5 min and 60 min after injection. ^7^ cART treatment interruption (ATI) starting from week 6 up to week 18. When there is an undetectable viral load at week 18, ATI is continued until week 30. Schedule according to template available from SPIRIT Statement,© 2013 Chan et al. [12]

Refer to Fig. 1 for a full overview of the study period, interventions and assessments. The study procedures according to the Standard Protocol Items: Recommendations for Interventional Trials (SPIRIT) Statement [12] are shown in Fig. 2 and are available as Additional file 1. The investigational medicinal product (IMP) in this study is iHIVARNA-01. In each center, their HIV treating physicians will inform patients about the phase-IIa clinical trial and confirm their eligibility. Those interested will receive a hardcopy/digital version of the patient information form (PIF) and are invited for the informed consent and screening visit, at most 8 weeks prior to eventual enrollment. The study will be discussed in detail and any questions will be answered. Specific attention applies for heterosexually active women: an effective method of contraception with their partner from 14 days prior to the first vaccination until 4 months after the last vaccination will be demanded. For heterosexually active men, using an effective method of contraception with their partner from the first day of vaccination until 4 months after the last vaccination will be demanded. The investigator repeats the former during follow-up visits in case of enrollment and actively questions the patients (or partner) adherence to these protocol-dictated life rules. Attention will be paid to risks of viral rebound syndrome and adequate measures for risk reduction of virus transmission during ATI will be discussed. An optional consideration period of up to 2 weeks is offered. The principal investigator (PI) (or their designated collaborator) will obtain written informed consent. Patients undergo a clinical examination and blood will be drawn. Demographic data, medical history and concomitant medication are recorded (refer to Fig. 2).

### Study procedures: enrollment and vaccinations

Review of screening data will be performed according to the inclusion and exclusion criteria. Female patients undergo a pregnancy test (repeated prior to each vaccination), which should return negative in order to continue with study participation. When the former criteria are met, a patient will be enrolled to the study. The investigator blindly assigns the patient to one of the treatment groups by allocating the next free randomization code for the clinical site to the patient. This randomization code list was generated by an independent statistician prior to start of the study (using R: A language and environment for statistical computing. R Foundation for Statistical Computing, Vienna, Austria). The label containing the randomization code was attached to the appropriate IMP at the central production facility in order to keep the blind for both the sponsor and investigators. The IMP is administered using ultrasound guidance by a radiologist sufficiently trained in ultrasound imaging and tissue biopsies. Injections will be given alternated between the right and left prominent inguinal lymph node. Patients will be monitored during the first hour after immunization by recording vital signs (pulse, blood pressure and respiratory rate) on the case record form (CRF), as well as any local reactions and systemic events (refer to Table 1). A diary card (refer to Tables 2 and 3) will be given to all the subjects, with written instructions and a verbal explanation to record local and systemic adverse events following 1 week after immunization. The diary is also used to track all medication (including over-the-counter ones) usage in the same period. Using a standardized letter, the treating general practitioner of the patient is informed of the study participation. Active reporting of eventual adverse events and/or the prescription of concomitant medication is demanded by informing the investigator without further due delay. Trial participation is mentioned in the electronic patient files, in such a way that other healthcare professionals are able to contact the study team for consultation or reporting of (serious) adverse events. The study team will hold a telephone call with patients 7 days (± 2 days) after the first immunization. This contact will inquire about adverse events, both local and systemic. Additional visits may be recommended at the discretion of the clinical and principal investigators. Assessments and vaccinations are repeated as described above at week 2 and week 4. Short visits are scheduled in between for blood draw to be able to answer the study’s objectives.

**Table 1:** Overview of assessments shortly after intranodal vaccination

Assessment	Options
Vaccination side	Left, right, not performed
Visual inspection of vaccination site	No abnormalities, erythema, blisters, other …
Echo-guided inspection of vaccination site	No abnormalities, pathological enlarged lymph node (… mm), other …
Time of vaccination	hh:mm
Vaccination in middle of lymph node	With certainty in middle, doubtful
Physical examination 5 and 50–70 min after procedure	Respiratory rate (RR) and pulse rate/min.

**Table 2:** Patient diary, general symptoms

	Grade 1: mild	Grade 2: moderate	Grade 3: severe	Grade 4: extreme
Chills/rigors	Mild hot/cold flushes requiring blanket or occasional painkillers/antipyretics	Limiting daily activity > 6 h or need regular painkillers/antipyretics	Uncontrollable shaking, treatment from doctor needed	Hospitalization
Malaise/tiredness	Normal activity reduced – not bad enough to go to bed	Fatigue such that 0.5 days in bed for 1 or 2 days	Fatigue such that is in bed all day or 0.5 day for more than 2 days	Hospitalization
General muscle aches	No limitation of activity	Muscle tenderness, limited activity, e.g., difficulty climbing stairs	Severe limitation, e.g., cannot climb stairs	Hospitalization
Headache	No treatment or responds to usual treatment like paracetamol	Occasional treatment like paracetamol needed	Strong pain killers needed (with prescription)	Hospitalization
Nausea	Intake maintained	Intake reduced at most 3 days	Minimal intake 3 days or more	Hospitalization
Vomiting	Less than 4 × a day or lasting less than 1 week	At least 4 x a day or lasting 1 week or more	Unable to keep any food of fluids down	Hospitalization

General symptoms are scored on a grade 1–4 scale at 12 h and 24 h post immunization. This is repeated every 24 h up to and including day 7

**Table 3:** Patient diary, local reactions

	Grade 1: mild	Grade 2: moderate	Grade 3: severe	Grade 4: extreme
Pain at injection site (including ache) but *not* skin discomfort	No treatment or responds to occasional treatment	Partial response to treatment	Need to use regular treatment to control pain	Hospitalization
Itching or irritation in the skin at site of injection	No treatment or responds well to treatment prescribed	Partial response to treatment	Need to use regular treatment to control symptoms	Hospitalization

Local reactions at the injection site (pain, itching) are scored on a grade 1–4 scale at 12 h and 24 h post immunization. This is repeated every 24 h up to and including day 7. In case there is any redness, fluid-filled blisters, blood-fluid blisters or hard swelling of the skin, the patient is instructed to measure and note their diameter in centimeters

### Study procedures: analytical treatment interruption (ATI), restart and final visit

Two weeks after the third vaccination (week-6 visit), cART will be interrupted. Female subjects will only be allowed interruption after a negative pregnancy test. In the visits at weeks 8, 10, 14, 18, 24 and 30, information on concomitant medication, a physical examination and laboratory tests (refer to Fig. 2) are obtained. At week 18, cART will be reinitiated unless a patient shows a stable undetectable viral load. Patients that have not resumed cART at week 30 (study end) because their plasma viral load remains undetectable and it is their preference not to re-initiate cART, should be closely monitored for plasma viral load and CD4+ cell counts on an (advised) 8-weekly base by their treating physicians.

### Study procedures: emergency and relevant considerations regarding ATI

In case of a medical urgency, revealing the study arm (i.e., unblinding) is possible by contacting unblinded pharmacy personnel at the sponsor’s pharmacy. This service is available 24 h a day. In case of accidental IMP breakage, loss, temperature excursions, etc., backup IMP is available at the study sites. The investigator can use a backup IMP vial by enquiring about the correct backup vial code at the pharmacy of the sponsor. Since this pharmacy has an unblinded list, the correct backup code can be issued.

ATI is needed in the search for an HIV cure [13]. During ATI patients will be monitored for adverse events of plasma viral rebound such as fever, fatigue, lymphadenopathy, pharyngitis, rash and/or weight loss. If the CD4+ T-cell counts drop below 50% of baseline level or below 350 cells/mL, an extra CD4+ T-cell count will be performed within 7 days. If the results are below 50% of baseline or below 350 cells/mL, cART will be re-initiated immediately. cART will also be re-initiated if the physician or investigator judges that this is in the best interest of the patient. A patient will prematurely discontinue the study in case of withdrawal of informed consent or if the investigator considers it to be in the best interest of the patient to withdraw. If a female participant becomes pregnant during the study, she will discontinue the study treatment (i.e., vaccination), but will be asked for consent for the pregnancy and delivery follow-up. Treatment will not be interrupted in these patients. In case a patient withdraws from the study, all efforts will be made to complete the week-30 assessments as the termination visit.

### Study procedures: reporting to authorities and temporary halt or suspension

According to the regulations of the European Union and as dictated by Dutch governmental law serious adverse events (SAEs) will be reported to the accredited Ethical Committee (EC) that approved the study within a period of maximum 15 days after the sponsor has first knowledge of the SAE. SAEs that results in death or are life-threatening will be reported within 7 days. Suspected unexpected serious adverse reactions (SUSARs) that have arisen in this trial (and, when applicable, other trials from the same sponsor or with the same study product) will be reported via an online portal. All SUSARs will be submitted in a line-listing twice a year. An annual safety report of SAEs and safety analysis will be submitted to the ECs and competent authorities. The sponsor will suspend the study if there is sufficient ground suggesting that continuation of the study will jeopardize patient health or safety. A Data Safety Monitoring Board (DSMB), an independent group of experts, advises the sponsor and will at least meet on four occasions prior to and during the study.

### Study endpoints

Refer to “Primary and secondary objectives” above.

### Statistical analysis including sample size calculation

Analysis will be performed using an intention-to-treat approach. Assuming a standard deviation of 0.5log^10^ in the change from baseline of cumulative frequencies of HTI-specific PBMC, a type 1 error of 5%, a power of 90%, as determined by a two-sided, non-parametric test (Mann-Whitney, 15% additional subjects), taking into account the weighting for case group ratio of more than 2:1, a sample of 40 in the HTI-TriMix arm and 15 in each control arm will allow to detect a difference of at least 0.7log^10^ between any HTI-TriMix arm and the controls arms, allowing for 10% of non-assessable patients.

## Discussion

In the challenging research field of HIV immunotherapy, we use the iHIVARNA-01 candidate vaccine in the search for cure. The strategy of the iHIVARNA consortium consists of a DC-based approach by intranodal administration of a newly developed HIV immunogen (HTI) and TriMix. Many of the immunotherapy strategies so far have relied on immunogens that cover regions of the virus that are frequently targeted by the immune system in the majority of chronic HIV-infected patients. These immune responses, even when enhanced, in general do not prevent disease progression [6, 14]. HTI is the result of a detailed investigation into effective antiviral T-cell responses and their fine specificity and aims to induce T-cell responses of high functional avidity and higher suppressive capacity towards vulnerable sites of HIV [9, 15].

In addition, co-stimulatory molecules are delivered in the formula of TriMix, consisting of CD40L, CD70 and TLR4a mRNA. TriMix was used in several trials, showing promising results in the field of tumor immunology [10, 16, 17] – including unpublished data; NCT 01302496, NCT01066390, NCT01530698. The rationale to provide extra immunomodulatory signals in the search for HIV cure was proven in a recent preclinical trial in SIV-infected macaques, where coincident Ad26/MVA vaccination and TLR7 agonist administration led to better immune stimulation of both the innate and cellular systems. This resulted in a delay in viral rebound and lower plasma viral load when cART was interrupted [18].

Concerning the administration of the vaccine, iHIVARNA was designed to target DCs. Promising results were achieved in former studies where autologous DCs were loaded ex vivo, with inactivated virus or electroporated with mRNA-encoding HIV proteins [19]. The current study thus circumvents this laborious DC isolation and handling in the laboratory by direct intranodal vaccination in the inguinal region. The use of naked mRNA bypasses recombinant vectors and their regulatory issues. However, this is only a proof-of-concept trial and studies currently are testing the stability of mRNA and targeted delivery, e.g., via chemical modifications and embedding in particles [20–23]. The goal will be to develop a vaccine that can be used from the fridge or even off the shelf (stored at room temperature) and that will target DCs after in-vivo administration.

The iHIVARNA phase-I, open-label, dose-escalation trial (NCT02413645) proved that this IMP is safe and well tolerated in HIV-1-infected patients on cART. The iHIVARNA phase IIa, is designed as a randomized, placebo-controlled, double-blinded trial. The three-arm design comprises (A) the full IMP (HTI and TriMix), (B) a placebo consisting of TriMix alone and (C) a placebo consisting of injection fluid. Arm B is important to be able to distinguish effects of general immune activation by TriMix from the effects of the full vaccine with the HIV-specific immunogen.

The trial will evaluate the safety and immunogenicity of iHIVARNA-01 in chronically HIV-infected patients under stable combined antiretroviral therapy and includes an analytical treatment interruption. Besides these primary endpoints, several secondary endpoints will be evaluated. The commonly known T-cell ELISPOT essay is extended with analysis of multiple cytokine expression after in-vitro stimulation [9, 24]. Also, an ELISPOT assay relying in-vitro re-stimulation with autologous B-cells expressing the vaccine immunogen is planned [25, 26]. This should give us a more comprehensive picture of the immune responses induced upon vaccination. An established virus suppression assay to test the in-vitro activity of patient-derived CD8+ T-cells on virus production by super-infected autologous CD4+ T-cells is available [27]. Beside the specific immune reactions, innate immune signatures will be monitored by transcriptome analysis, as this has shown informative in a previous trial [28].

Control of in-vivo viral replication will be studied after analytical treatment interruption and different endpoint approaches will be considered, i.e., time to viral rebound, levels of pVL and the proportion of patients with undetectable viremia after 12 weeks of treatment interruption. Effects on the viral reservoir will be measured with various assays during the immunization period, including ultra-sensitive plasma virus polymerase chain reaction (PCR) and PCR-based approaches to measure cell associated viral RNA and DNA. In case of viral rebound, the evolution of the virus will be followed with deep sequencing to evaluate mechanisms of immune escape and/or a potential sieve effect on the rebounding viral populations after the immunotherapy [29]. These results may indicate either an advantageous effect, if the viral and proviral loads decrease and the virus mutates to a less fit population. On the other hand, we might observe escape from the vaccine-induced immune pressure, without a gross effect on viral burden.

To summarize, the trial has a comprehensive set of assays that will be used to establish the effects of the immunotherapy in vitro and in vivo. If this candidate would be able to obtain the functional cure in at least a proportion of patients it could be complementary to cART or even replace cART and would improve the overall care and cost of HIV-1 infected patients.

## Trial status

Recruiting patients since 27 March 2017.

## Additional file

Additional file 1: Standard Protocol Items: Recommendations for Interventional Trials (SPIRIT) 2013 Checklist populated for for iHIVARNA phase IIa. (DOC 120 kb)

## Abbreviations

AIDS: Acquired immunodeficiency syndrome
ATI: Analytical treatment interruption
cART: Combined antiretroviral therapy
CRF: Case record form
DC: Dendritic cell
DSMB: Data Safety Monitoring Board
EC: Ethical Committee
HIV: Human immunodeficiency virus
HTI: HIVACAT T-cell immunogen
iHIVARNA-01: Investigational medicinal product under development: HTI-TriMix
PIF: Patient information form
SAEs: Serious adverse events
SUSARs: Suspected unexpected serious adverse reactions
WFI: Water for injection

## Footnotes

1 During the revision process of this paper, it became apparent that the study product iHIVARNA-01 contains an error in that the RNA sequence contained by mistake a second start codon in front of the HTI immunogen coding sequence. This error is likely to influence the expression of the HTI protein, from the mRNA vaccine. Even though the degree to which this impacted the expression of HTI remains unclear, the results of the preclinical trial show that there is sufficient expression for the induction of an immunogen-specific T-cell response in mice.

## Authors’ information

Not provided.

## References

[1] WHO. HIV/AIDS data and statistics: World Health Organization; 2017. Available from: http://www.who.int/hiv/data/en/. Accessed 11 May 2017.

[2] Phillips AN, Eron JJ, Bartlett JA, Rubin M, Johnson J, Price S (1996). HIV-1 RNA levels and the development of clinical disease. North American Lamivudine HIV Working Group. AIDS.

[3] Siliciano JD, Kajdas J, Finzi D, Quinn TC, Chadwick K, Margolick JB (2003). Long-term follow-up studies confirm the stability of the latent reservoir for HIV-1 in resting CD4+ T cells. Nat Med.

[4] WHO. Prevent HIV, test and treat all—WHO support for country impact. Progress report 2016: WHO; 2016. https://www.who.int/hiv/pub/progressreports/2016-progress-report/en/. Accessed 11 May 2017.

[5] Leal L, Lucero C, Gatell JM, Gallart T, Plana M, Garcia F (2017). New challenges in therapeutic vaccines against HIV infection. Expert Rev Vaccines.

[6] Barouch DH, Deeks SG (2014). Immunologic strategies for HIV-1 remission and eradication. Science.

[7] Carcelain G, Autran B (2013). Immune interventions in HIV infection. Immunol Rev.

[8] Garcia F, Climent N, Guardo AC, Gil C, Leon A, Autran B (2013). A dendritic cell-based vaccine elicits T cell responses associated with control of HIV-1 replication. Sci Transl Med.

[9] Mothe B, Hu X, Llano A, Rosati M, Olvera A, Kulkarni V (2015). A human immune data-informed vaccine concept elicits strong and broad T-cell specificities associated with HIV-1 control in mice and macaques. J Transl Med.

[10] Bonehill A, Tuyaerts S, Van Nuffel AM, Heirman C, Bos TJ, Fostier K (2008). Enhancing the T-cell stimulatory capacity of human dendritic cells by co-electroporation with CD40L, CD70 and constitutively active TLR4 encoding mRNA. Mol Ther.

[11] AETC. HIV Classification: CDC and WHO Staging Systems: AIDS Education & Training Center Program; 2014. [Available from: https://aidsetc.org/guide/hiv-classification-cdc-and-who-staging-systems. Accessed 11 May 2017.

[12] Chan AW, Tetzlaff JM, Gotzsche PC, Altman DG, Mann H, Berlin JA (2013). SPIRIT 2013 explanation and elaboration: guidance for protocols of clinical trials. BMJ.

[13] Calin R, Hamimi C, Lambert-Niclot S, Carcelain G, Bellet J, Assoumou L (2016). Treatment interruption in chronically HIV-infected patients with an ultralow HIV reservoir. AIDS.

[14] Richman DD, Margolis DM, Delaney M, Greene WC, Hazuda D, Pomerantz RJ (2009). The challenge of finding a cure for HIV infection. Science.

[15] Mothe B, Llano A, Ibarrondo J, Zamarreno J, Schiaulini M, Miranda C (2012). CTL responses of high functional avidity and broad variant cross-reactivity are associated with HIV control. PLoS One.

[16] Van Lint S, Renmans D, Broos K, Goethals L, Maenhout S, Benteyn D (2016). Intratumoral delivery of TriMix mRNA results in T-cell activation by cross-presenting dendritic cells. Cancer Immunol Res.

[17] Van Lint S, Wilgenhof S, Heirman C, Corthals J, Breckpot K, Bonehill A (2014). Optimized dendritic cell-based immunotherapy for melanoma: the TriMix-formula. Cancer Immunol Immunother.

[18] Borducchi EN, Cabral C, Stephenson KE, Liu J, Abbink P, Ng'ang'a D (2016). Ad26/MVA therapeutic vaccination with TLR7 stimulation in SIV-infected rhesus monkeys. Nature.

[19] Allard SD, De Keersmaecker B, de Goede AL, Verschuren EJ, Koetsveld J, Reedijk ML (2012). A phase I/IIa immunotherapy trial of HIV-1-infected patients with Tat, Rev and Nef expressing dendritic cells followed by treatment interruption. Clin Immunol.

[20] Kauffman KJ, Dorkin JR, Yang JH, Heartlein MW, DeRosa F, Mir FF (2015). Optimization of lipid nanoparticle formulations for mRNA delivery in vivo with fractional factorial and definitive screening designs. Nano Lett.

[21] Oberli MA, Reichmuth AM, Dorkin JR, Mitchell MJ, Fenton OS, Jaklenec A (2017). Lipid nanoparticle assisted mRNA delivery for potent cancer immunotherapy. Nano Lett.

[22] Tavernier G, Andries O, Demeester J, Sanders NN, De Smedt SC, Rejman J (2011). mRNA as gene therapeutic: how to control protein expression. J Control Release.

[23] Van Lint S, Heirman C, Thielemans K, Breckpot K (2013). mRNA: from a chemical blueprint for protein production to an off-the-shelf therapeutic. Hum Vaccin Immunother.

[24] Ruiz-Riol M, Llano A, Ibarrondo J, Zamarreno J, Yusim K, Bach V (2015). Alternative effector-function profiling identifies broad HIV-specific T-cell responses in highly HIV-exposed individuals who remain uninfected. J Infect Dis.

[25] Dock J, Hultin L, Hultin P, Elliot J, Yang OO, Anton PA (2017). Human immune compartment comparisons: optimization of proliferative assays for blood and gut T lymphocytes. J Immunol Methods.

[26] Lambotte O, Pollara J, Boufassa F, Moog C, Venet A, Haynes BF (2013). High antibody-dependent cellular cytotoxicity responses are correlated with strong CD8 T cell viral suppressive activity but not with B57 status in HIV-1 elite controllers. PLoS One.

[27] Van Gulck E, Vlieghe E, Vekemans M, Van Tendeloo VF, Van De Velde A, Smits E (2012). mRNA-based dendritic cell vaccination induces potent antiviral T-cell responses in HIV-1-infected patients. AIDS.

[28] de Goede AL, Andeweg AC, van den Ham HJ, Bijl MA, Zaaraoui-Boutahar F, van IJcken WF (2015). DC immunotherapy in HIV-1 infection induces a major blood transcriptome shift. Vaccine.

[29] de Goede AL, van Deutekom HW, Vrancken B, Schutten M, Allard SD, van Baalen CA (2013). HIV-1 evolution in patients undergoing immunotherapy with Tat, Rev, and Nef expressing dendritic cells followed by treatment interruption. AIDS.

