# Correction to: iHIVARNA phase IIa, a randomized, placebo-controlled, double-blinded trial to evaluate the safety and immunogenicity of iHIVARNA-01 in chronically HIV-infected patients under stable combined antiretroviral therapy

**DOI:** 10.1186/s13063-019-3940-0

**Published:** 2019-12-13

**Authors:** Wesley de Jong, Joeri Aerts, Sabine Allard, Christian Brander, Jozefien Buyze, Eric Florence, Eric van Gorp, Guido Vanham, Lorna Leal, Beatriz Mothe, Kris Thielemans, Montse Plana, Félipe Garcia, Rob Gruters, on behalf of the iHIVARNA consortium

**Affiliations:** 1 Department of Viroscience, Erasmus MC, Room Ee-1726, P.O. Box 2040, 3000 CA Rotterdam, The Netherlands; 2 Laboratory of Molecular and Cellular Therapy, Vrije Universiteit Brussel, Brussels, Belgium; 3 Department of Internal Medicine and Infectious Diseases, Universitair Ziekenhuis Brussel, Brussels, Belgium; 4 Infectious Diseases Unit, IrsiCaixa AIDS Research Institute, Hospital Germans Trias i Pujol, Badalona, Spain; 5 Institució Catalana de Recerca i Estudis Avançats (ICREA), Barcelona, Spain; 6 AELIX Therapeutics, Parc Científic de Barcelona, Barcelona, Spain; 7 University of Vic – Central University of Catalonia (UVic-UCC), Vic, Spain; 8 Virology Unit, Department of Biomedical Sciences, Institute of Tropical Medicine and, Antwerp, Belgium; 9 Department of Biomedical Sciences, University of Antwerp, Antwerp, Belgium; 10 Institut d’Investigacions Biomèdiques August Pi i Sunyer (IDIBAPS), Villarroel, 170, 08036 Barcelona, Spain; 11 Infectious Diseases Unit, Hospital Clínic, Villarroel, 170, 08036 Barcelona, Spain

**Correction to: Trials**

**https://doi.org/10.1186/s13063-019-3409-1**

Following publication of the original article [1], we have been notified that the end note would need to be adjusted.

Originally published note:

- During the revision process of this paper, it became apparent that the study product iHIVARNA-01 contains an error in that the RNA sequence contained by mistake a second start codon in front of the HTI immunogen coding sequence. This error is likely to influence the expression of the HTI protein, from the mRNA vaccine. Even though the degree to which this impacted the expression of HTI remains unclear, the results of the preclinical trial show that there is sufficient expression for the induction of an immunogen-specific T-cell response in mice.

Corrected end note:

- After completing the phase I and phase II trial, it became apparent that the study product iHIVARNA-01 contains an error in that the RNA sequence contained by mistake a second start codon upstream of the HTI immunogen coding sequence. This error is likely to influence the expression of the HTI protein, from the mRNA vaccine. The degree to which this impacted the expression of HTI remains unclear. The results of the preclinical study showed an induction of an immunogen specific T cell response in mice which could not be correlated with known HTI expression as the expression level of the HTI peptide has not been assessed. Similarly, in the clinical studies, the level of expression of the immunogen was not quantified.
